# Tick-Borne Pathogens in Wild Cervids from Poland: A Molecular Survey on *Anaplasma phagocytophilum* and Piroplasms

**DOI:** 10.3390/ani16172747

**Published:** 2026-09-02

**Authors:** Emily Sel, Giulia Libertini, Fabrizio Bertelloni, Ewa Bilska-Zając, Agnieszka Jodełko, Valentina Virginia Ebani, Lisa Guardone

**Affiliations:** 1 Department of Veterinary Science, University of Pisa, Viale Delle Piagge 2, 56124 Pisa, Italy; emily.sel@phd.unipi.it (E.S.); giulia.libertini@vet.unipi.it (G.L.); fabrizio.bertelloni@unipi.it (F.B.); valentina.virginia.ebani@unipi.it (V.V.E.); 2 Department of Parasitology and Invasive Diseases, Bee Diseases and Aquatic Animal Diseases, National Veterinary Research Institute, 24-100 Puławy, Poland; ewa.bilska@piwet.pulawy.pl; 3 Department of Bacteriology and Bacterial Animal Diseases, National Veterinary Research Institute, 24-100 Puławy, Poland; agnieszka.jodelko@piwet.pulawy.pl

**Keywords:** ticks, wildlife, ruminants, *Anaplasma* spp., *Theileria* spp.

## Abstract

Tick-borne diseases (TBDs) are important infections affecting animal health, and some are zoonotic. Understanding the distribution of TBDs in wildlife is important to evaluate the role of wild animals in the maintenance and spread of tick-borne pathogens (TBPs) in natural ecosystems. This study investigates the occurrence of selected TBPs, including *Anaplasma phagocytophilum* and piroplasms (*Babesia* spp. and *Theileria* spp.), in three widespread wild cervids from Poland: red deer, fallow deer, and roe deer. Ninety spleen tissue samples (30 per species) were collected and analyzed by endpoint PCR and sequencing. DNA of *A. phagocytophilum* was detected in 54.6% of the samples, mainly in red deer (75.9%), followed by fallow deer (46.4%) and roe deer (41.4%). Piroplasm DNA was found in 30% of the samples, with the highest prevalence in red deer (47%), followed by fallow deer (27%) and roe deer (17%). Sequencing analysis showed the presence of *Theileria capreoli* and identified two distinct genotypes: “elaphi-CE1”, associated with red deer and fallow deer, and “capreoli-CE1”, associated with roe deer. The results update the epidemiology of TBPs in Poland, supporting the role of cervids as potential reservoirs of *A. phagocytophilum* and *T. capreoli*, confirming the need for surveillance in wildlife. Further studies are needed to investigate the distribution of *T*. *capreoli* genotypes across Europe.

## Simple Summary

Tick-borne diseases affect both animal and human health, and wild animals can play an important role in maintaining and spreading the pathogens that cause these infections. To better understand this role, we investigated the presence of selected tick-borne pathogens in three common wild deer species from Poland: red deer, fallow deer, and roe deer. Spleen samples from 90 animals were tested for the bacteria *Anaplasma phagocytophilum* and for piroplasms, blood parasites. More than half of the animals carried *A. phagocytophilum*, with the highest prevalence found in red deer. Piroplasms were detected in 30% of the samples, mainly in red deer. In detail, genetic analysis only revealed the presence of *Theileria capreoli*, and in particular of two distinct *T. capreoli* genotypes, each associated with different deer species, whereas no *Babesia* species were identified. These findings improve our understanding of the distribution of tick-borne pathogens in Polish wildlife and highlight the potential role of wild cervids as natural reservoirs of these microorganisms, some of which can affect humans. Continued surveillance of wildlife populations is important to monitor the circulation of tick-borne pathogens and to better understand their ecology and potential impact on animal and public health across Europe.

## 1. Introduction

Vector-borne diseases (VBDs) pose a significant threat to animal and public health worldwide. Among these, tick-borne diseases (TBDs) have assumed increasing importance over the past decades. Ticks are, in fact, competent vectors for a wide range of pathogenic microorganisms, including viruses, bacteria, and parasites. The transmission of these pathogens is strongly influenced by the interaction between the environment, the vector and the host. In particular, climate change, ecosystem alterations, and the increase in wildlife populations have facilitated the expansion of ticks and intensified interactions between wildlife, domestic animals, and humans, contributing to the spread of tick-borne pathogens (TBPs) [1,2,3].

In this context, wildlife plays a key role as a reservoir and vector for numerous TBPs and should be monitored in the framework of a One Health surveillance approach. Among wild animals, wild ruminants are of ecological importance in the maintenance and spread of tick populations. In Europe, species such as red deer (*Cervus elaphus)*, fallow deer *(Dama dama*), and roe deer (*Capreolus capreolus*) are widely distributed and frequently exposed to blood-feeding arthropods, thereby contributing to the circulation of pathogens in natural ecosystems [4,5,6,7]. Among the most relevant TBPs found in European cervids are bacteria such as *Anaplasma phagocytophilum* and blood protozoa such as *Babesia* spp. and *Theileria* spp.

*Anaplasma phagocytophilum,* a Gram-negative obligate intracellular bacterium of the order *Rickettsiales*, is the etiological agent of granulocytic anaplasmosis, which may affect several animal species, mainly horses and dogs, as well as humans (human granulocytic anaplasmosis, HGA). HGA is relatively common in the USA, where over 5500 cases were diagnosed in 2019, while only about 300 cases were reported in Europe in the same year. It is still unclear whether this discrepancy is related to different epidemiological dynamics in Europe or whether the disease is underdiagnosed or underreported [8]. Domestic ruminants may develop clinical disease, whereas wild ruminants, which are considered important reservoir hosts, are generally asymptomatic [5]. *A. phagocytophilum* is associated with a variety of tick species that facilitate its spread across different geographic regions [9]; however, *Ixodes ricinus*, the most widespread European tick species, is considered the principal vector in Europe [5].

Piroplasms, protozoan parasites belonging to the phylum Apicomplexa and primarily to the genera *Babesia* and *Theileria*, affect a wide range of domestic and wild animals. Different species have been reported in wild ungulates in Europe [10,11,12]. Some of these species may also affect domestic ruminants [12,13,14], and others are recognized as zoonotic pathogens [15]. Among these, *Babesia venatorum*, associated with roe deer, has been identified as responsible for human cases [12]. In wildlife, these infections are generally considered asymptomatic, and wild animals are therefore regarded as reservoir hosts that contribute to the maintenance and circulation of these parasites in the natural ecosystem [7,10]. However, occasional clinical manifestations have been reported, suggesting that piroplasm infections can, under certain circumstances, also cause disease in wildlife [16,17]. Even fatal cases due to *B. capreoli* and *B. venatorum* have been reported in captive reindeer [12]. Different tick genera, such as *Ixodes*, *Rhipicephalus*, *Haemaphysalis*, *Hyalomma, Amblyomma*, and *Dermacentor* contribute to the transmission of piroplasms [7,10].

Despite the widespread distribution of cervids in Europe, knowledge regarding the circulation of TBPs in these species remains incomplete in some geographic areas. In Poland, where the cervid population is consistent and widely distributed, molecular data on the presence of TBPs are dated or limited, particularly for fallow deer [17,18,19,20,21]. All this considered, the aim of this study was to assess the presence of selected TBPs, specifically *A. phagocytophilum* and piroplasms, in three species of wild cervids in Poland, using molecular methods. The obtained data aim to contribute to TBDs surveillance with a One Health approach.

## 2. Materials and Methods

**Figure 1:**
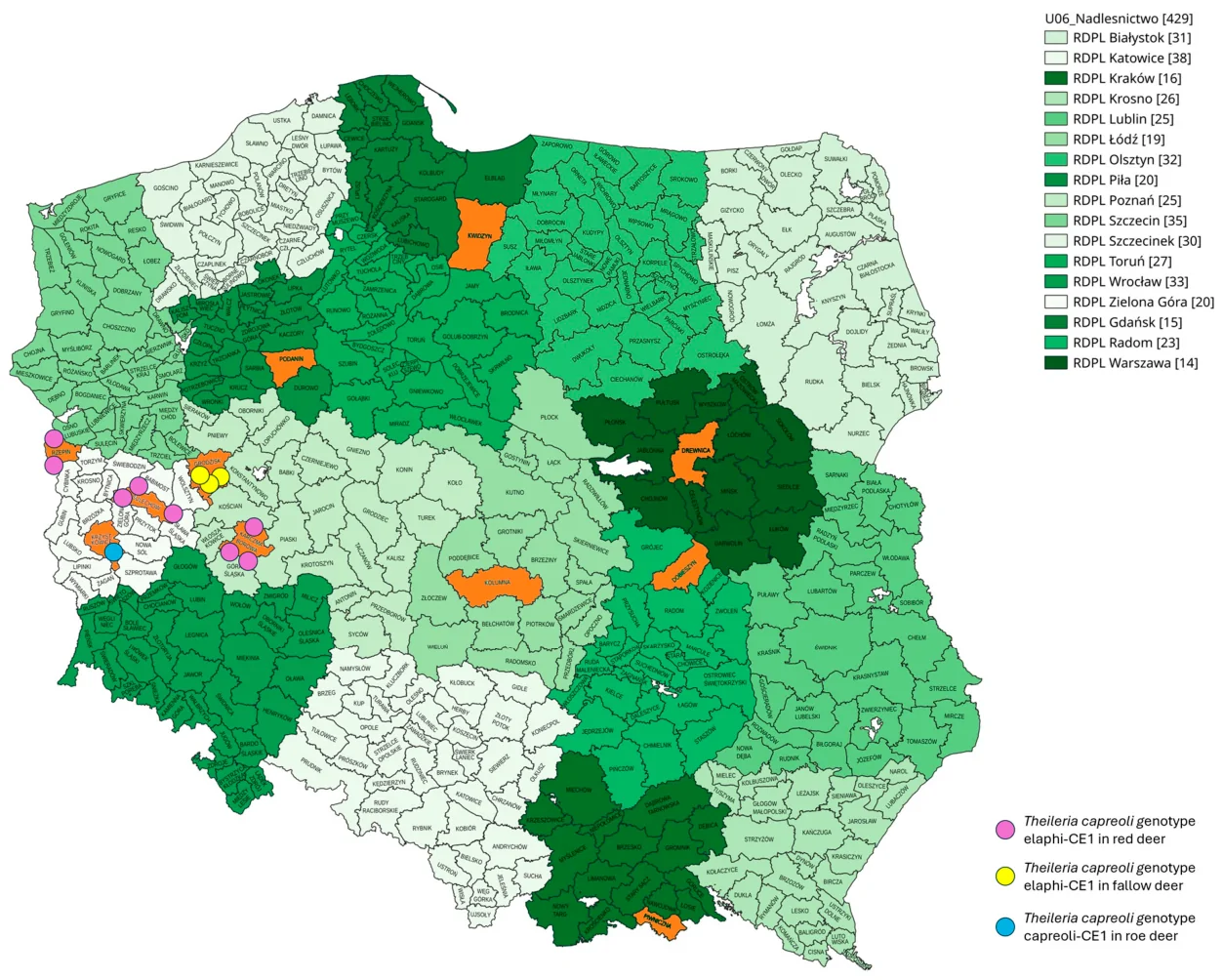
Map of Poland showing the distribution of forest districts (Nadleśnictwo) within the State Forests National Forest Holding and the distribution of *Theileria capreoli* genotypes in the three cervid species. Green areas represent all forest districts, classified by their respective Regional Directorates of State Forests (RDLP), while orange areas indicate the districts where animal sampling was conducted. The numbers in square brackets indocate the total number of forest districts (Nadleśnictwo) [429] in Poland and the number of forest districts in each RDLP.

Biological samples (spleen tissue) were collected from regularly hunted wild ruminants during the 2023 hunting season in various forest districts across different regions of Poland (Figure 1), as part of the project “Wild animals as a reservoir of infectious and invasive pathogens dangerous to human and livestock health” (grant from the Minister of Agriculture and Rural Development of Poland, Agreement No. DŻW.zlf.890.6.2023). A total of 90 animals were sampled, belonging to three species of wild ruminants: 30 red deer (*C. elaphus*), 30 roe deer (*C. capreolus*), and 30 fallow deer *(D. dama*). All samples were stored at −20 °C until the time of analysis.

### 2.1. DNA Extraction

Genomic DNA was extracted from approximately 10 mg of spleen tissue per sample using the commercial QIAamp DNA Mini Kit (Qiagen GmbH, Hilden, Germany) following the manufacturer’s instructions. Extracted DNA was subsequently sent to the Department of Veterinary Sciences at the University of Pisa, Italy, where molecular analyses were performed.

### 2.2. PCR Amplifications

The presence of *A. phagocytophilum* and *Babesia*/*Theileria* spp. DNA was investigated using endpoint PCRs with different primers and cycling conditions, as detailed in Table 1.

The PCR reactions were set up in a final volume of 40 μL containing 6 μL of template DNA, 1 μL of 10 μM primers, 8 μL reaction buffer, 0.5 μL of Taq DNA polymerase (Wonder Taq, Eurofins Genomics, Ebersberg, Germany), and 23.5 μL of PCR-grade water. Positive (known positive samples confirmed by sequencing and deposited in GenBank under accession numbers ON729315—*A. phagocytophilum*—and PZ805159—*Babesia* sp.) and negative controls (sterile molecular grade water) were included in each run to ensure the reliability of the results. PCR products were visualized by using a UV transilluminator (Benchmark Accuris™ UV Transilluminator, Sayreville, NJ, USA) after electrophoresis migration (100 V per 40 min) on 2% agarose gel stained with GelRed^®^ Nucleic Acid Gel Stain (Biotium, Fremont, CA, USA) in 1× standard tris-acetate-EDTA (TAE) buffer. SharpMass™ 100 Plus Ladder (Euroclone, Milan, Italy) was used as a molecular weight marker. Amplicons showing the expected fragment size were considered positive.

### 2.3. Sequencing

A subset of PCR products that tested positive for piroplasms were sent to an external laboratory (Eurofins Genomics, Ebersberg, Germany) for Sanger sequencing [24,25]. The obtained sequences were manually edited using FinchTV (Geospiza) and subsequently compared with those in the GenBank database using the BLAST (Basic Local Alignment Search Tool, program version: BLASTN 2.17.0+) algorithm on http://www.ncbi.nlm.nih.gov/BLAST (accessed on 3 June 2026) to determine their identity and match known piroplasms species. Sequencing analyses were also requested to confirm *A. phagocytophilum* amplicons.

### 2.4. Data Analysis

The prevalence of positive cases for each host species (*C. capreolus*, *C. elaphus*, *D. dama*) and the corresponding 95% confidence intervals were calculated. A comparison between overall differences among species and forest districts was conducted by Pearson’s or Fisher chi square tests.

## 3. Results

Overall, 58 animals (64.4%) were positive for at least one target pathogen, in detail, 24 (80.0%; 95% CI: 62.7–90.5) red deer, 18 (60.0%; 95% CI: 42.3–75.4) fallow deer, and 16 (53.3%; 95% CI: 36.1–69.8) roe deer. The presence of *A. phagocytophilum* DNA was observed in 47 samples, corresponding to an overall prevalence of 54.6% (86 samples were tested for this pathogen). The highest prevalence was observed in red deer (75.9%), followed by fallow deer (46.4%) and roe deer (41.4%) (Table 2). Prevalence differed significantly among cervid species (χ^2^ = 8.09, *p* = 0.018), while differences among forest districts were not statistically significant (χ^2^ = 14.89, *p* = 0.192).

Piroplasm DNA (*Babesia/Theileria* spp.) was identified in 27 samples, corresponding to an overall prevalence of 30% (95% CI). The distribution of positive results was not uniform among the different host species (χ^2^ = 6.66, *p* = 0.036), with the highest prevalence observed in red deer (46.7%), followed by fallow deer (26.7%) and roe deer (16.7%) (Table 2). Piroplasm prevalence also differed markedly among forest districts (χ^2^ = 37.66, *p* < 0.001). The highest prevalence was observed in Sulechów (6/7, 85.7%) and Grodzisk (8/11, 72.7%), whereas no positive animals were detected in Dobieszyn, Drewnica, Kwidzyń, Podanin, or Kolumna. Coinfections of *A. phagocytophilum* and piroplasms were observed in 16 animals (17.8%), mainly in red deer (n = 12, 40.0%), followed by fallow deer (n = 3, 10.0%) and roe deer (n = 1, 3.3%). Detailed results for each sample on animal species, forest district, and administrative regions (voivodeships) regarding positivity for *A. phagocytophilum* and piroplasms are reported in Table S1.

### Sequencing Results and Presence of Different Genotypes

Six (two for each animal species) randomly chosen *A. phagocytophilum* amplicons were sequenced to confirm the PCR result. All sequences had 100% homology with *A. phagocytophilum* sequences registered in GenBank. Two sequences were deposited in the GenBank database under accession numbers PZ546420 and PZ546421, respectively.

Following the identification of piroplasm PCR-positive samples, a subsample of 13 amplicons from the three examined host species were sequenced, including 8 from red deer, 4 from fallow deer, and 1 from roe deer. Sequences analysis revealed a high degree of homology (100%) with *T. capreoli*, confirming the presence of this species in all three species of wild ruminants analyzed. In detail, the roe deer sample showed a 100% match with *T. capreoli* (GenBank accession no. MH085202.1), as did the eight samples from red deer. Regarding fallow deer samples, three sequences showed a 100% match with a different sequence of *T. capreoli* (MW531681.1), while one sequence, which was of lower quality, allowed for identification only at the genus level as *Theileria* sp. Three representative sequences (one per species) were deposited in the GenBank database under accession numbers PZ760896–PZ760898.

Moreover, the BLAST analysis of the obtained *T. capreoli* sequences revealed the presence of two distinct genotypes, previously described in Hungary and designated as “capreoli-CE1” (reference deposited sequence KY308178.1) and “elaphi-CE1” (reference deposited sequence KY308179.1) [11]. In our results, the distribution of these genotypes differed according to the host species: the “capreoli-CE1” genotype was detected exclusively in roe deer, while the “elaphi-CE1” genotype was identified exclusively in red deer and fallow deer.

## 4. Discussion

The results obtained in this study confirm the circulation of TBPs in a non-negligible rate of the examined wild ruminants from Poland. The pathogens detected have a zoonotic potential (*A. phagocytophilum*) and may be transmitted to livestock (*T. capreoli*), underlining the relevance of wildlife monitoring in a One Health approach. The spleen was chosen as target tissue considering that it is easier to sample by hunters in dead animals compared to other matrices such as blood and also considering that it is widely used in similar international studies [4,7,10,11,17,21].

The high prevalence of *A. phagocytophilum* found (54.62%) shows the widespread circulation of this microorganism in wild ruminants in the study area and suggests the key epidemiological role of these species in maintaining the natural cycle of the infection. This result is consistent with findings from studies conducted in Europe, including Poland, which found prevalences ranging from 20% to up to 90% in relation to the geographical area and confirmed cervids as important reservoirs of *A. phagocytophilum* [4,5,26,27,28]. The prevalence observed also highlights the potential risk for humans. Indeed, previous epidemiologic studies in Europe suggested that for forestry workers, hunters, veterinarians, and farmers with a tick-bite history and living in endemic areas, an increased risk exists [8]. Besides the impact on human health, anaplasmosis is a relevant veterinary concern. In Europe, in particular, *A. phagocytophilum* strains appear to be particularly virulent. Infected cattle show high fever, anemia, leukopenia, and thrombocytopenia, and reduced productivity. Normally, no symptoms are observed in wild ruminants, with some exceptions that may present weight loss, anorexia, and apathy. Rare fatal cases following *A. phagocytophilum* infection have been described in sheep and in roe deer and moose in Norway [as reported in 5].

Few epidemiological data are available on this pathogen for wild ruminants in Poland (Table 3). The significantly higher prevalence observed in red deer in the present study suggests that this species may represent one of the principal reservoir hosts in the studied ecosystem. This is particularly relevant considering that in a recent study, it was demonstrated that genetic variants of *A. phagocytophilum* isolated from red deer in Poland can be pathogenic to humans [5]. Together with roe deer, red deer constitute the largest group of game animals in Poland [5]. Compared with roe deer and fallow deer, red deer generally occupy larger home ranges and make greater use of extensive forest habitats. In addition, they represent important hosts for adult *Ixodes ricinus*. Consequently, they may experience more frequent exposure to infected ticks, facilitating the maintenance of both tick populations and pathogen circulation within wildlife communities.

With regard to piroplasms, our findings highlight the presence of the species *T. capreoli* in wild ruminants from the study area. Moreover, we documented the occurrence of two genetically distinct but closely related genotypes: the “elaphi-CE1” genotype, identified only in red deer and fallow deer, and the “capreoli-CE1” genotype, associated with roe deer. Unfortunately, sequencing was not successful for a part of the positive samples. This represents the main limitation of the study, as it is not possible to rule out the circulation of other species or genotypes in the analyzed population.

A similar epidemiological picture has already been described in Hungary [11], where *T. capreoli* was found predominantly in roe deer (43.1%), followed by red deer (41.7%) and fallow deer (23.5%), while no positive results were detected in the other examined species, such as wild boars and water buffalo [11]. Thus, the prevalences observed in red deer and fallow deer in the present study are similar, although slightly higher, to those reported in the Hungarian study, while the prevalence in roe deer appears lower. The observed distribution could suggest species-related differences in exposure or susceptibility. Regarding the distribution of the two genotypes, Hornok et al. [11] found “capreoli-CE1” exclusively in roe deer, while “elaphi-CE1” was identified in red deer, fallow deer, and a mouflon, in full agreement with the results obtained in the present study. This consistency across different geographic areas reinforces the hypothesis that the distribution of the two genotypes is not a random phenomenon, but rather represents a stable biological characteristic, likely linked to evolutionary differences among the host species: indeed, the roe deer belongs to the Capreolinae subfamily, while red deer and fallow deer are part of the Cervinae group [29]. Although we acknowledge that the number of animals investigated in the study is limited and that further studies on a larger number of samples and on a wider geographical area are needed, the repeated observation of the same host–genotype associations in geographically distant populations suggests that this pattern is biologically stable rather than environmentally driven.

The occurrence of *T. capreoli* has previously been reported in red deer from Poland, with prevalence values reaching approximately 88% [17]. Information on the occurrence of *T. capreoli* in roe deer and fallow deer in Poland remains limited (Table 3). The results obtained in the present survey showed epidemiological differences across forest districts for piroplasms. Although such differences should be interpreted cautiously due to the small sample sizes of some districts, they suggest strong geographical heterogeneity in piroplasm circulation. The highest prevalence was observed in Sulechów and Grodzisk, whereas no positive animals were detected in Dobieszyn, Drewnica, Kwidzyń, Podanin, or Kolumna. The two forest districts with the highest prevalence are situated in western Poland, where extensive and well-connected forest complexes support abundant populations of wild cervids. Continuous forest cover facilitates animal movements, increases contact rates among hosts, and provides suitable habitats for ixodid ticks. Such environmental conditions may favor the maintenance and circulation of tick-borne pathogens. In contrast, no positive animals were detected in Dobieszyn, Drewnica, Kwidzyń, Podanin, or Kolumna, which represent more heterogeneous landscapes, including fragmented forests, agricultural mosaics, and in the case of Drewnica, suburban forests surrounding Warsaw. Greater habitat fragmentation, higher proportions of agricultural land, and increased anthropogenic pressure may reduce wildlife connectivity and consequently limit pathogen transmission opportunities. Nevertheless, landscape structure alone is unlikely to fully explain the observed spatial heterogeneity. Other ecological factors, including host population density, age structure, tick abundance, seasonal variation, and local microclimatic conditions, probably interact to determine pathogen circulation. The recent expansion of *Haemaphysalis concinna* into western Poland may also have contributed to the higher prevalence observed in this region [30]. This tick species has previously been shown to harbor *T. capreoli* DNA in Central Europe [11,31]. Although its epidemiological importance in Poland remains insufficiently understood, its increasing distribution may contribute to the higher prevalence observed in the western forest districts.

At the European level, *T. capreoli* is widely distributed among wild cervids, with prevalence rates varying across different countries and ecological regions. This species has been reported in red deer in Italy [32] and in several cervid species in Portugal [33], with prevalence values ranging from approximately 0.3% to nearly 88%. Red deer and fallow deer appear to contribute significantly to the persistence of *T. capreoli* in the environment, while roe deer can be considered as a sentinel species, as they are often the first to show the presence of tick-borne parasites in a given area [10,31,34]. Although *T. capreoli* is not considered zoonotic, it may affect domestic animal species, as it was recently reported in sheep in Portugal [35]. Interestingly, this record occurred in an area where this species had previously been reported in red deer and wild boar [33], suggesting potential transmission between wild and domestic hosts.

In addition to *T. capreoli,* numerous other species of piroplasms belonging to the genera *Babesia* and *Theileria* have been described in European wild ruminants, such as *B. bigemina*, *B. capreoli*, *B. divergens*, *B. microti*, *B. odocoilei*, *B. ovis*, *Babesia* sp. MO1, *B. venatorum* (formerly *Babesia* sp. EU1), *Theileria* sp. 3185/02, *Theileria* sp. OT3, and *Theileria* sp. ZSTO4 [10,12,14,36]. Among these, as mentioned, *B. venatorum* is known to have a zoonotic potential [12].

The epidemiological differences observed among the different European countries could be attributed to various factors, including local ecological differences, population densities and vector distribution. In Germany, for example, a predominance of *I. ricinus* [37] was found on roe deer, a finding consistent with the presence of *Babesia* sp. EU1 and *Babesia odocoilei*-like in this host species [38]. In contrast, in Hungary, *Haemaphysalis concinna* has been shown to play a significant role in cervids, especially red deer and roe deer, and has been found to carry *T. capreoli* DNA [11,31]. As mentioned, recent studies have confirmed its presence in western forest areas in Poland [30].

In addition to the composition of tick populations, climatic and geographic factors can also influence the distribution of TBPs, as pathogen circulation is shaped not only by the presence of competent vectors, but also by the interaction between climate, habitat composition, wildlife density, and host community structure. For example, Poland and Hungary, although both located in Central Europe, are characterized by a more continental climate than Germany, with colder winters and warmer, drier summer. Together with differences in vegetation cover and wildlife host distribution, these conditions may influence tick communities and consequently TBP circulation. Such differences are even more evident when comparing Central Europe with Mediterranean regions, such as Spain, where the warmer and drier climate has been associated with a higher occurrence of *Babesia* spp. in cervids [10,39]. Similarly, environmental variability in the Alpine region, including Switzerland and northern Italy, may affect tick distribution and piroplasm circulation in wild ruminants [12,36,40].

## 5. Conclusions

Overall, the results obtained in this molecular survey highlight the relevant role of wild cervids in the epidemiological dynamics of TBPs in Poland, a context for which previous epidemiological data are scarce or limited. In particular, their relevance is supported by the presence of *A. phagocytophilum* and *T. capreoli,* while *Babesia* spp. was not detected in the samples analyzed. *Anaplasma phagocytophilum* has a zoonotic potential, while *T. capreoli* may be transmitted to livestock, underlining the need for integrating wildlife in a One Health TBP surveillance approach. Furthermore, the results obtained help reinforce the hypothesis of an association between specific genotypes of *T. capreoli* and certain host species.

Taken together, our findings suggest that the epidemiology of TBPs in Polish cervids is shaped by the interaction between host species, vector ecology, and local environmental conditions rather than by a single ecological factor. Future studies integrating molecular surveillance with ecological and spatial analyses will be necessary to better understand these complex relationships. Health monitoring of wild cervids is essential for both wildlife management and the assessment of epidemiological risks for human and domestic animals. However, further studies involving a larger number of samples and geographic areas will be necessary to fully clarify the relative contribution of different wild hosts to the circulation of these pathogens.

## Figures and Tables

**Table 1:** Primers, target gene, fragment length, and PCR amplification conditions used for the molecular detection of *Anaplasma phagocytophilum* and *Babesia*/*Theileria* spp.

Molecular Assay	Primers	Target Gene	Fragment Length (bp)	PCR Conditions	Reference
PCR for *Anaplasma* *phagocytophilum* (first PCR)	GE3a: CACATGCAAGTCGAACGGATTATTC GE10r: TTCCGTTAAGAAGGATCTAATCTCC	16SrRNA	932	95 °C for 1 min 55 °C for 1 min 72 °C for 2 min (40 cycles)	[22]
(Nested PCR)	GE9f: AACGGATTATTCTTTATAGCTTGCT GE2: GGCAGTATTAAAAGCAGCTCCAGG		546		
PCR for *Babesia* and *Theileria* species	Mic1: GTCTTGTAATTGGAATGATGG Mic2: CCAAAGACTTTGATTTCTCTC	18SrRNA	560	95 °C for 30 s 53 °C for 30 s 72 °C for 1 min (40 cycles)	[23]

**Table 2:** Number of analyzed samples, positive samples, and prevalence (with 95% confidence intervals) of *Anaplasma phagocytophilum* and piroplasms (*Babesia*/*Theileria* spp.) in wild ruminants in Poland.

Species	Samples Positive for Piroplasms/Analyzed Samples	Prevalence of Piroplasms (95% IC)	Samples Positive for *Anaplasma phagocytophilum*/Analyzed Samples	Prevalence of *Anaplasma phagocytophilum* (95% IC)
Red deer (*Cervus elaphus*)	14/30	46.7% (30.2–63.9)	22/29 ^a^	75.9% (57.9–87.8)
Fallow deer (*Dama dama*)	8/30	26.7% (14.2–44.4)	13/28 ^a^	46.4% (29.5–64.2)
Roe deer (*Capreolus capreolus*)	5/30	16.7% (7.3–33.6)	12/29 ^a^	41.4% (25.5–59.3)
Overall	27/90	30.0% (20.8–40.6)	47/86 ^a^	54.6% (44.2–64.7)

^a^ Due to technical reasons (lack of extracted DNA) fewer samples (totally 86) were tested for *A. phagocytophilum.*

**Table 3:** Occurrence of *Anaplasma phagocytophilum*, *Babesia* spp. and *Theileria* spp. in wild ruminants in Poland as reported in the literature (since 2005).

Pathogen	Animal Species	Prevalence (n Pos/n Examined)	Reference
*B. divergens*	Deer (roe/red deer)	24.4% (20/82)	[19]
*Theileria* spp.		11% (9/82)	
*Theileria* spp.	Red deer	88% (36/41)	[17]
*Babesia* spp.	Roe deer	30.4% (42/138)	[20]
	Red deer	2% (1/50)	
*Theileria* spp.	Roe deer	24.6% (34/138)	
	Red deer	84% (42/50)	
*B. venatorum*	Roe deer	16.6% (2/12)	[21]
*B. divergens*		16.6% (2/12)	
*B. capreoli*		66.7% (8/12)	
*B. capreoli*	Roe deer	14,3% (1/7)	[18]
*A. phagocytophilum*	Roe deer Red deer	Not reported	[5]
*A. phagocytophilum*	Roe deer Red deer Fallow deer	Not reported	[26]

## Data Availability

The original contributions presented in this study are included in the article/Supplementary Materials. Further inquiries can be directed to the corresponding author.

## Supplementary Materials

The following supporting information can be downloaded at: https://www.mdpi.com/article/10.3390/ani16172747/s1, Table S1: Detailed results for piroplasms and *Anaplasma phagocytophilum* for animal species, forest district, and administrative regions (voivodeships).

## Abbreviations

The following abbreviations are used in this manuscript:

BLAST	Basic Local Alignment Search Tool
CI	Confidence interval
DNA	Deoxyribonucleic acid
PCR	Polymerase chain reaction
RDLP	Regional Directorates of State Forests
TBDs	Tick-borne diseases
TBPs	Tick-borne pathogens
VBDs	Vector-borne diseases
