# Supplementary material for: Tick-Borne Pathogens in Wild Cervids from Poland: A Molecular Survey on Anaplasma phagocytophilum and Piroplasms
Source: Animals (Basel). 2026 Sep 2;16(17):2747. doi: 10.3390/ani16172747 (PMC13565653; doi:10.3390/ani16172747)
Supplement: Supplementary file 1 [file animals-16-02747-s001.zip › animals-4505533-supplementary.pdf]

Table S1. Detailed results for piroplasms and *Anaplasma phagocytophilum* for animal species, forest district, and administrative regions (*voivodeships*).

Sample ID	Animal species	Forest district	Region (Voivodeship(s)	*Babesia/Theileria* spp. PCR Results	*Anaplasma phagocytophilum* PCR Results	Co-positivity
521A (nr1)	Roe deer	Drewnica	Mazowieckie	neg	pos	
521A (nr2)	Roe deer	Drewnica	Mazowieckie	neg	pos	
521A (nr3)	Roe deer	Drewnica	Mazowieckie	neg	pos	
521A (nr4)	Roe deer	Drewnica	Mazowieckie	neg	neg	
521A (nr5)	Roe deer	Drewnica	Mazowieckie	neg	pos	
521A (nr6)	Roe deer	Drewnica	Mazowieckie	neg	neg	
521A (nr7)	Roe deer	Drewnica	Mazowieckie	neg	neg	
521A (nr8)	Roe deer	Drewnica	Mazowieckie	neg	pos	
521A (nr9)	Roe deer	Drewnica	Mazowieckie	neg	neg	
518A (nr70)	Roe deer	Kwidzyń	Pomorskie	neg	pos	
518A (nr71)	Roe deer	Kwidzyń	Pomorskie	neg	pos	
518A (nr72)	Roe deer	Kwidzyń	Pomorskie	neg	neg	
518A (nr73)	Roe deer	Kwidzyń	Pomorskie	neg	neg	
518A (nr74)	Roe deer	Kwidzyń	Pomorskie	neg	neg	
518A (nr75)	Roe deer	Kwidzyń	Pomorskie	neg	neg	
518A (nr76)	Roe deer	Kwidzyń	Pomorskie	neg	neg	
575A (nr 184)	Roe deer	no data	no data	pos	/	
575A (nr 185)	Roe deer	no data	no data	pos	neg	
606A (nr 203)	Roe deer	Krzystkowice	Lubuskie	pos	pos	X
606A (nr 204)	Roe deer	Krzystkowice	Lubuskie	neg	pos	
606A (nr 205)	Roe deer	Krzystkowice	Lubuskie	neg	neg	
606A (nr 210)	Roe deer	Krzystkowice	Lubuskie	neg	neg	
606A (nr 211)	Roe deer	Krzystkowice	Lubuskie	neg	pos	
606A (nr 212)	Roe deer	Krzystkowice	Lubuskie	neg	neg	
606A (nr 213)	Roe deer	Krzystkowice	Lubuskie	neg	pos	
606A (nr 214)	Roe deer	Krzystkowice	Lubuskie	neg	neg	
606A (nr 215)	Roe deer	Krzystkowice	Lubuskie	neg	pos	
606A (nr 216)	Roe deer	Krzystkowice	Lubuskie	pos	neg	

611A (nr228)	Roe deer	Piwniczna	Małopolskie	pos	neg	
611A (nr229)	Roe deer	Piwniczna	Małopolskie	neg	neg	
517A (nr 38)	Red deer	Rzepin	Lubuskie	pos	pos	X
517A (nr 39)	Red deer	Rzepin	Lubuskie	neg	neg	
517A (nr 40)	Red deer	Rzepin	Lubuskie	neg	pos	
517A (nr 41)	Red deer	Rzepin	Lubuskie	neg	pos	
517A (nr 42)	Red deer	Rzepin	Lubuskie	neg	neg	
515A (nr30)	Red deer	Rzepin	Lubuskie	pos	pos	X
515A (nr31)	Red deer	Rzepin	Lubuskie	neg	neg	
516A (nr35)	Red deer	Sulechów	Lubuskie	neg	pos	
516A (nr36)	Red deer	Sulechów	Lubuskie	pos	pos	X
516A (nr 37)	Red deer	Sulechów	Lubuskie	pos	/	
525A (nr 78)	Red deer	Rzepin	Lubuskie	neg	pos	
525A (nr 79)	Red deer	Rzepin	Lubuskie	pos	pos	X
524A (nr 26)	Red deer	Sulechów	Lubuskie	pos	pos	X
524A (nr 27)	Red deer	Sulechów	Lubuskie	pos	pos	X
524A (nr 28)	Red deer	Sulechów	Lubuskie	pos	pos	X
524A (nr 29)	Red deer	Sulechów	Lubuskie	pos	pos	X
570A (nr 139)	Red deer	Karczma Borowa	Wielkopolskie	pos	pos	X
570A (nr 140)	Red deer	Karczma Borowa	Wielkopolskie	pos	pos	X
570A (nr 141)	Red deer	Karczma Borowa	Wielkopolskie	neg	pos	
570A (nr 142)	Red deer	Karczma Borowa	Wielkopolskie	neg	neg	
570A (nr 143)	Red deer	Karczma Borowa	Wielkopolskie	pos	pos	X
570A (nr 144)	Red deer	Karczma Borowa	Wielkopolskie	neg	pos	
570A (nr 145)	Red deer	Karczma Borowa	Wielkopolskie	neg	pos	
570A (nr 146)	Red deer	Karczma Borowa	Wielkopolskie	neg	neg	
570A (nr 147)	Red deer	Karczma Borowa	Wielkopolskie	neg	pos	
570A (nr 148)	Red deer	Karczma Borowa	Wielkopolskie	neg	neg	
570A (nr 149)	Red deer	Karczma Borowa	Wielkopolskie	neg	pos	
570A (nr 150)	Red deer	Karczma Borowa	Wielkopolskie	pos	neg	
570A (nr 151)	Red deer	Karczma Borowa	Wielkopolskie	neg	pos	
570A (nr 152)	Red deer	Karczma Borowa	Wielkopolskie	pos	pos	X

619A (nr 252)	Fallow deer	Grodzisk	Wielkopolskie	pos	neg	
619A (nr 253)	Fallow deer	Grodzisk	Wielkopolskie	neg	pos	
619A (nr 254)	Fallow deer	Grodzisk	Wielkopolskie	pos	neg	
619A (nr 255)	Fallow deer	Grodzisk	Wielkopolskie	pos	pos	X
619A (nr 256)	Fallow deer	Grodzisk	Wielkopolskie	neg	pos	
619A (nr 257)	Fallow deer	Grodzisk	Wielkopolskie	pos	neg	
621A (nr 258)	Fallow deer	Podanin	Wielkopolskie and kujawsko-pomorskie	neg	neg	
621A (nr 259)	Fallow deer	Podanin	Wielkopolskie and kujawsko-pomorskie	neg	pos	
621A (nr 261)	Fallow deer	Podanin	Wielkopolskie and kujawsko-pomorskie	neg	pos	
662A (nr 262)	Fallow deer	Podanin	Wielkopolskie and kujawsko-pomorskie	neg	neg	
662A (nr 263)	Fallow deer	Podanin	Wielkopolskie and kujawsko-pomorskie	neg	pos	
663A (nr264)	Fallow deer	Podanin	Wielkopolskie and kujawsko-pomorskie	neg	neg	
663A (nr265)	Fallow deer	Podanin	Wielkopolskie and kujawsko-pomorskie	neg	neg	
663A (nr266)	Fallow deer	Podanin	Wielkopolskie and kujawsko-pomorskie	neg	neg	
627A (nr 277)	Fallow deer	Dobieszyn	Mazowieckie	neg	pos	
627A (nr 278)	Fallow deer	Dobieszyn	Mazowieckie	neg	neg	
627A (nr 279)	Fallow deer	Dobieszyn	Mazowieckie	neg	neg	
627A (nr 280)	Fallow deer	Dobieszyn	Mazowieckie	neg	pos	
627A (nr 281)	Fallow deer	Dobieszyn	Mazowieckie	neg	pos	
645A (nr 297)	Fallow deer	Grodzisk	Wielkopolskie	neg	neg	
645A (nr 298)	Fallow deer	Grodzisk	Wielkopolskie	pos	pos	X
645A (nr 299)	Fallow deer	Grodzisk	Wielkopolskie	pos	/	
645A (nr 300)	Fallow deer	Grodzisk	Wielkopolskie	pos	pos	X
645A (nr 301)	Fallow deer	Grodzisk	Wielkopolskie	pos	/	

650A (nr 302)	Fallow deer	Dobieszyn	Mazowieckie	neg	neg	
650A (nr 303)	Fallow deer	Dobieszyn	Mazowieckie	neg	neg	
650A (nr 304)	Fallow deer	Dobieszyn	Mazowieckie	neg	neg	
692A (nr 382)	Fallow deer	Kolumna	Łódzkie	neg	pos	
692A (nr 383)	Fallow deer	Kolumna	Łódzkie	neg	neg	
692A (nr 384)	Fallow deer	Kolumna	Łódzkie	neg	pos	
